# Temporal trajectory of biofluid markers in Parkinson’s disease

**DOI:** 10.1038/s41598-021-94345-8

**Published:** 2021-07-20

**Authors:** Min Seok Baek, Myung Jun Lee, Han-Kyeol Kim, Chul Hyoung Lyoo

**Affiliations:** 1 Department of Neurology, Wonju Severance Christian Hospital, Yonsei University Wonju College of Medicine, Wonju, Gangwon do Republic of Korea; 2 Department of Neurology, Pusan National University Hospital, Pusan National University School of Medicine and Biomedical Research Institute, Gudeok-ro 179, Seo-gu, Busan, 49241 Republic of Korea; 3 Department of Neurology, Gangnam Severance Hospital, Yonsei University College of Medicine, 20 Eonjuro 63-gil, Gangnam-gu, Seoul, Republic of Korea

**Keywords:** Biomarkers, Neurology, Parkinson's disease

## Abstract

Full dynamics of biofluid biomarkers have been unknown in patients with Parkinson’s disease (PD). Using data from 396 PD patients and 182 controls in the Parkinson's Progression Markers Initiative (PPMI) database, we estimated long-term temporal trajectories of CSF α-synuclein (α-syn), amyloid-β (Aβ), total tau (t-tau), phosphorylated tau (p-tau) and serum neurofilament light chain (NfL) by integrating function between the baseline levels and annual changes. At baseline, PD patients showed lower CSF α-syn, Aβ, t-tau and p-tau levels than those of the controls. In all PD patients, CSF α-syn and Aβ decreased in a negative exponential pattern before the onset of motor symptoms, whereas CSF t-tau and p-tau, and serum NfL increased. Patients with cognitive impairment exhibited faster decline of Aβ and α-syn and faster rise of t-tau, p-tau and NfL, when compared to those without. Similarly, low Aβ group showed earlier decline of α-syn, faster rise of t-tau, p-tau and NfL, and faster decline of cognitive performances, when compared to high Aβ group. Our results suggest that longitudinal changes in biomarkers can be influenced by cognitive impairment and Aβ burden at baseline. PD patients with Aβ pathology may be associated with early appearance of α-synuclein pathology, rapid progression of axonal degeneration and neurodegeneration, and consequently greater cognitive decline.

## Introduction

About 80% of Parkinson’s disease (PD) patients become demented in their clinical course with variable time intervals between the onset of motor symptoms and deterioration of cognition^1–3^. Pathologically, greater amount of cortical and limbic Lewy bodies (LB), amyloid-β (Aβ) plaque and neurofibrillary tangles are related with the development of dementia in PD^4,5^. CSF biomarkers for each pathological protein may reflect the pathological burden in brain, and thereby, long-term changes in CSF biomarkers may be related with both pathological and clinical progression in PD.

However, several studies observed inconsistent results of longitudinal changes in CSF biomarkers. Some exhibited an increase in CSF α-synuclein (α-syn) along with the disease progression and an association of α-syn with further deterioration of motor and cognitive deficits^6,7^, while others reported a decrease in CSF α-syn^8,9^. Likewise, contradictory results of the longitudinal changes in CSF Aβ_1-42_ were reported^10,11^. These studies observed the changes occurring in relatively short time period (from 18 months to 4 years) at different time points of disease course, and therefore, were insufficient for providing full dynamics of CSF biomarkers.

In the present study, using the longitudinal data for the biomarkers in the Parkinson's Progression Markers Initiative (PPMI) study, we estimated long-term temporal trajectories of CSF α-syn, Aβ_1-42_, total tau (t-tau), phosphorylated tau (p-tau) and serum neurofilament light chain (NfL) by integrating the function between baseline level and annual change rates^12,13^. In addition, we investigated effects of cognitive impairment and low CSF Aβ_1-42_ level on the temporal trajectories of CSF biomarkers.

## Results

### Demographics, clinical measurement and biomarker levels at baseline

Demographic characteristics, clinical measurement and biomarker levels at baseline are summarized in Table 1. In total, 396 PD and 182 control subjects were included in the analyses. PD patients had lower CSF Aβ_1-42_ (β = − 106.9, SE 39.9, *p* = 0.008), α-syn (β = − 183.1, SE 65.0, *p* = 0.005), t-tau (β = − 24.3, SE 5.7, *p* < 0.001) and p-tau levels (β = − 2.7, SE 0.6, *p* < 0.001) than those of the controls, while the serum NfL levels did not exhibit a significant difference. In addition, PD patients showed worse cognitive performance in MoCA and HVLT delayed recall (MoCA, β = − 1.3, SE 0.2, *p* < 0.001; HVLT, β = − 3.9, SE 1.0, *p* < 0.001). Lower CSF α-syn and t-tau levels showed a trend of association with higher MDS-UPDRS III total scores at baseline, however, generalized linear models did not reach statistical significance (CSF α-syn, β = − 7.15, SE 3.85, *p* = 0.065; CSF t-tau, β = − 0.62, SE 0.34, *p* = 0.069).

**Table 1:** Baseline demographics, biomarker levels and cognitive outcomes in PD and control subjects.

	Control	Total PD	High CSF Aβ_1-42_	Low CSF Aβ_1-42_	PDCU	PDCI
n	182	396	274	122	311	85
Age	60.6 ± 11.5	61.7 ± 9.7	61.5 ± 9.7	62.1 ± 9.8	60.91 ± 9.7	64.5 ± 9.5*
Sex (M:F)	115: 67	264: 132	181: 93	83: 39	204: 107	60:25:00
Education years	15.9 ± 2.9	15.5 ± 3.0	15.6 ± 2.9	15.5 ± 3.1	15.7 ± 3.0	15.1 ± 2.7
Disease duration	n.a	2.0 ± 2.0	2.0 ± 2.0	2.0 ± 2.1	2.0 ± 1.9	2.1 ± 2.4
APOE ε4 + (n)	45/166	98/360	52/198*	46/64	80/282	18/78
MDS-UPDRS III	n.a	21.3 ± 9.0	21.3 ± 8.8	21.4 ± 9.4	20.7 ± 8.7	23.5 ± 9.6*
H&Y stage (I/II/III)	n.a	169/225/2	119/154/1	50/71/1	136/174/1	33/51/1
CSF Aβ_1-42_	1022.6 ± 502.1	916.0 ± 414.8*	1094.7 ± 373.7*	514.6 ± 109.1	913.5 ± 416.3	925.0 ± 412.0
CSF α-syn	1701.3 ± 770.7	1528.7 ± 678.7*	1693.0 ± 686.9*	1173.0 ± 503.5	1512.3 ± 646.7	1588.7 ± 786.5
CSF t-tau	192.9 ± 80.4	170.5 ± 57.9	181.1 ± 53.9	144.2 ± 59.2	169.8 ± 56.5	173.0 ± 63.3
CSF p-tau	17.6 ± 8.5	15.0 ± 5.4*	15.5 ± 5.0*	13.3 ± 6.0	14.8 ± 5.2	15.6 ± 6.1
Serum NfL	12.4 ± 9.9	13.7 ± 11.8	13.7 ± 13.0	13.6 ± 8.7	13.5 ± 12.9	14.3 ± 6.3
MoCA	28.2 ± 1.1	27.1 ± 2.3*	27.1 ± 2.3	27.4 ± 2.2	28.1 ± 1.3	23.6 ± 1.7*
HVLT-DR	48.8 ± 11.0	44.8 ± 11.1*	44.9 ± 11.0	44.5 ± 11.4	46.2 ± 10.8	39.6 ± 10.7*
LNS	10.9 ± 2.6	10.6 ± 2.6	10.5 ± 2.7	10.7 ± 2.4	10.8 ± 2.6	9.5 ± 2.4*

Data are presented as mean ± SD. *P < 0.05 for total PD *vs.* controls, low CSF Aβ_1-42_ *vs.* high CSF Aβ_1-42_, PDCU *vs.* PDCI.

*PDCU* cognitively unimpaired PD; *PDCI* cognitively impaired PD patients; *APOE* apolipoprotein E; *MDS-UPDRS III* Movement Disorder Society sponsored Unified Parkinson’s Disease Rating Scale part III; *H&Y stage* Hoehn & Yahr stage; *Aβ*_*1-42*_ amyloid-β_1-42_; *α-syn* α-synuclein; *t-tau* total tau; p-tau = phosphorylated tau; *NfL* neurofilament light chain; *MoCA* total scores of Montreal Cognitive Assessment; *HVLT-DR* delayed recall score in Hopkins Verbal Learning Test; *LNS* total scores of Letter-Number Sequencing test; *n.a.* not available.

311 PD patients showed MoCA total scores greater than 25 at baseline and were classified as PDNC. Although the PDCI group was older age and showed worse parkinsonian motor deficits at baseline than the PDNC, there was no clear difference in baseline biomarker levels between the two groups. According to the baseline CSF Aβ_1-42_, 274 PD patients were classified as high Aβ_1-42_ group and remaining 122 patients as low Aβ_1-42_. PD patients with low Aβ_1-42_ showed higher frequency of APOE ε4 allele and lower baseline CSF α-syn and p-tau than those with high CSF Aβ_1-42_. Serum NfL and cognitive performances did not show significant differences between the two groups.

**Figure 1:**
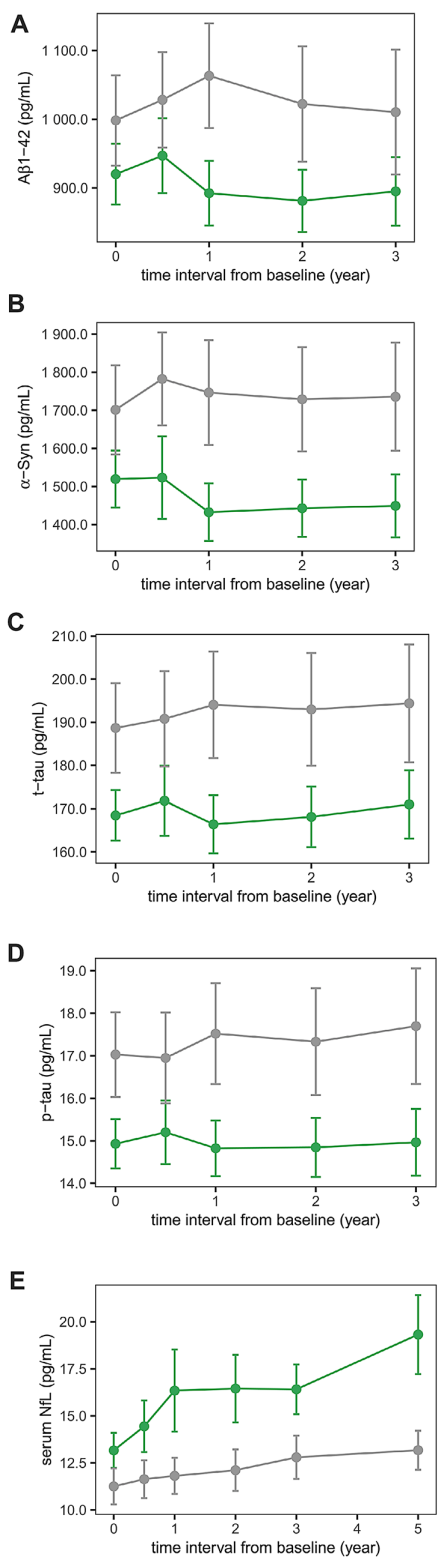
Measured biomarker levels in the PPMI study. Dots represent mean values of biomarkers at each follow-up visit. Error-bars denote 95% confidence interval (green lines and dots = PD patients; gray lines and dots = controls). Figures are illustrated using ggplot2 and patchwork packages in R software. *Aβ*_*1-42*_ amyloid-β_1-42_; *α-syn* α-synuclein; *t-tau* total tau; *p-tau* phosphorylated tau; *NfL* neurofilament light chain.

During the follow-up periods, entire PD patients showed an overall trend of decrease in CSF α-syn and Aβ_1-42_, whereas serum NfL were increased. CSF t-tau and p-tau levels remained almost constant from the baseline to 3rd year of follow-up (Fig. 1). In group comparisons using linear mixed effect models using age, sex and groups (PD and controls) as fixed effects and subjects as random effect, PD patients showed lower CSF α-syn (estimate − 285.9, SE 56.8, *p* < 0.001), Aβ_1-42_ (estimate − 143.2, SE 37.0, *p* < 0.001), t-tau (estimate − 28.4, SE 5.7, *p* < 0.001) and p-tau (estimate − 3.1, SE 0.6, *p* < 0.001) than control subjects, whereas serum NfL did not exhibit a significant difference during study period.

### Temporal trajectories in total PD patients

**Figure 2:**
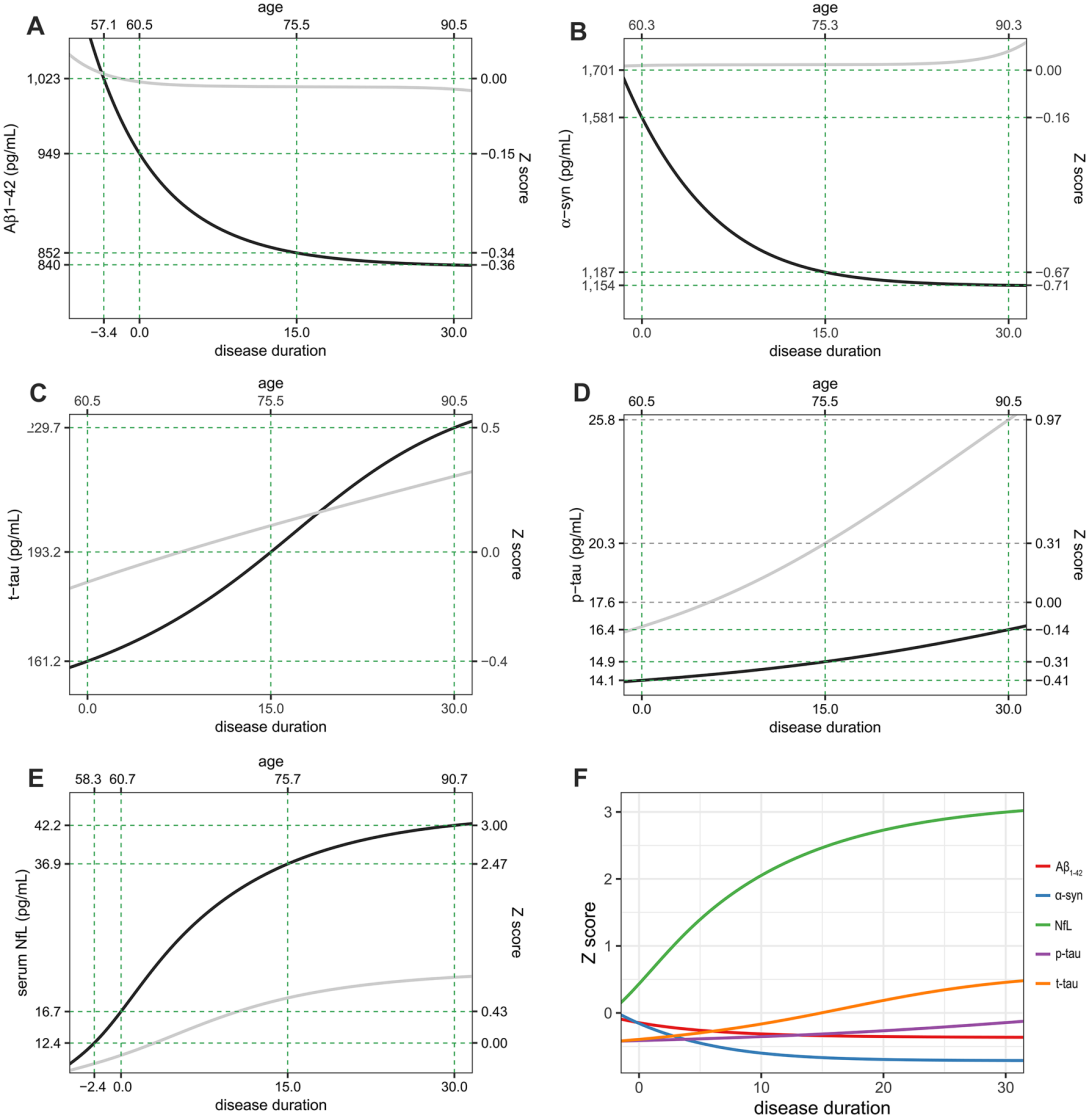
Estimated trajectories of biofluid markers in PD (black line) and control groups (gray line) as function of time. The temporal trajectories were acquired by modified Euler’s method for solving the first order differential equation. Temporal trajectories of CSF Aβ_1-42_ and α-syn show negative exponential pattern with suspicious plateau (**A**,**B**), while those of the CSF t-tau and p-tau, and serum NfL show steady increase pattern (**D**) or sigmoid appearance (**C**,**E**). Z score change in serum NfL was the largest within biofluid markers (**F**). Figures are illustrated using ggplot2 and patchwork packages in R software. *Aβ*_*1-42*_ amyloid-β_1-42_; *α-syn* α-synuclein; *t-tau* total tau; *p-tau* phosphorylated tau; *NfL* neurofilament light chain.

The restricted cubic spline function for baseline values vs. annual changes in CSF Aβ_1-42_ showed lower estimated annual changes in PD group than control subjects. Estimated annual changes were positive under 839 pg/mL of baseline baseline Aβ_1-42_ and 1014.1 pg/mL in controls, and then converted into negative at higher levels (Supplementary Fig. S1A). As a result, temporal trajectory of CSF Aβ_1-42_ in PD group initially showed a rapid decline from the premotor phase until about 5 years after the onset of motor symptoms and then slowly approached a plateau. Overall changes in CSF Aβ_1-42_ during the 30 years after the onset was estimated as − 12% (Fig. 2A). In contrast, trajectory in control group exhibited stable CSF Aβ_1-42_ values during estimated period.

Restricted cubic spline curves for CSF α-syn showed similar pattern to those for Aβ_1-42_. Estimated annual changes continuously decreased along with the increase of baseline level, and converted into negative above 1,152 pg/mL of baseline in PD group and 1,715 pg/mL in control group (Supplementary Fig. S1B). Temporal trajectory of CSF α-syn showed a decline from the premotor phase, and then slowly approached a plateau. Overall changes in CSF α-syn during the 30 years after the onset was estimated as − 26.9% whereas trajectory of CSF in α-syn control group showed nearly steady level (Fig. 2B).

Estimated annual changes of CSF t-tau level in control group were slightly positive against baseline values, however, PD patients were estimated to exhibit negative annual changes over 242 pg/mL of baseline t-tau level (Supplementary Fig. S1C). In the estimated trajectories, PD patients had a lower CSF t-tau (161.2 pg/mL) at disease duration 0 than controls (184.6 pg/mL). Estimated temporal trajectories of CSF t-tau in PD and control groups exhibited increase of biomarker levels over time, however, CSF t-tau levels in PD group became higher about 19 years after motor onset compared to controls. Estimated CSF t-tau in PD group increased by 42% during 30 years of motor stage (Fig. 2C).

PD patients exhibited a lower CSF p-tau level at motor onset than that of the controls and the mean annual changes in CSF p-tau was close to zero (0.06 pg/mL/year) and smaller than those in controls (0.31 pg/mL/year; Supplementary Fig. S1D). Although the temporal trajectory of CSF p-tau showed a steady increase even after 30 years after the onset (Fig. 2D), it did not rose above the Z-score 0.

Finally, the curve for the estimated annual changes of serum NfL against baseline levels in PD group showed an inverted-U shape with a peak at 18.8 pg/mL of baseline. Restricted cubic spline curve for control group exhibited similar pattern, however estimated annual changes at peak (0.61 pg/mL/year at 13.4 pg/mL) was lower than that of PD patients (2.0 pg/mL/year). (Supplementary Fig. S1E). Temporal trajectory of serum NfL thereby showed a rapid increase compared to controls, and the increase rate was slowly decreased after 10 years after the onset. Compared to the estimated level at the onset, serum NfL in PD group showed an increase of 153% over the 30 years of symptomatic period (Fig. 2E), which was larger change than CSF Aβ_1-42_, α-syn, t-tau and p-tau. (Fig. 2F).

### Influence of cognitive impairment at baseline on temporal trajectories of biomarkers

Linear mixed effect models using groups (PDCU and PDCI), disease duration, and interaction between groups and disease duration and subjects as random effect showed significant effect of interaction between disease duration and groups in progressions of CSF Aβ_1-42_, α-syn, t-tau and p-tau levels. Compared to PDCU group, patients with cognitive impairment at baseline exhibited slower decrement of CSF Aβ1-42 (estimate 22.94, SE 9.52, *p* = 0.016) and α-syn (estimate 63.3, SE 18.9, *p* < 0.001) and faster increase of t-tau (estimate 4.41, SE 1.29, *p* = 0.001) and p-tau (estimate 0.34, SE 0.10, *p* = 0.001; Supplementary Table S1).

**Figure 3:**
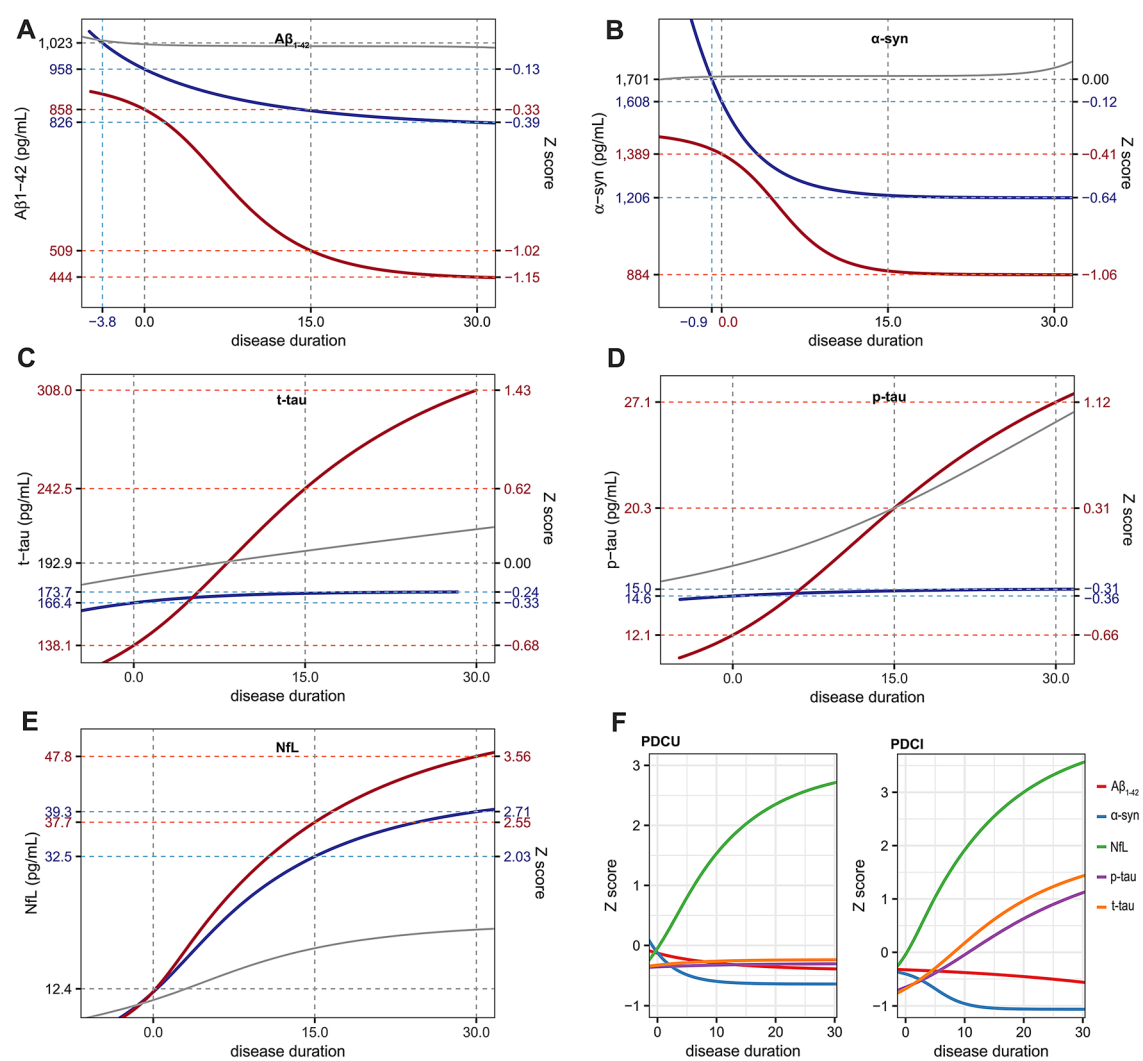
Temporal trajectories of biomarkers in PD patients with and without cognitive impairment at baseline. PD patients with cognitive impairment (red lines) show greater change in all five biomarkers (**A**: Aβ_1-42_, **B**: α-syn, **C**: t-tau, **D**: p-tau, **E**: NfL) across the disease course than those without (blue lines). NfL showed the most remarkable changes both in PDCU and PDCI groups (**F**). Gray lines represent temporal trajectories in control group. Figures are illustrated using ggplot2 and patchwork packages in R software. *Aβ*_*1-42*_ amyloid-β_1-42_; *α-syn* α-synuclein; *t-tau* total tau; *p-tau* phosphorylated tau; *NfL* neurofilament light chain.

In the estimated trajectory, PDCI group showed a rapid decline of CSF Aβ_1-42_ and, while the PDCU group did not. During the 30 years of symptomatic period, PDCI group showed much greater change in CSF Aβ_1-42_ (− 48%) than PDCU group (− 14%; Fig. 3A). In the trajectories of CSF α-syn, both PDCU and PDCI groups approached plateau about 15 years from motor onset. However, PDCI group had lower α-syn level at motor onset and exhibited greater reduction (− 36%) during the 30 years of motor phase than PDCU patients (− 25%; Fig. 3B).

PDCU subset had a lower CSF t-tau level than PDCI group. In the estimated trajectories, PDCU group showed a little increment of t-tau (increase of 4.4% during 30 years after motor onset), whereas estimated t-tau levels in PDCI group showed a consistent rise (increase of 123.0% during 30 years after the onset). Estimated CSF t-tau levels of PDCI group at motor onset was lower than those of PDCU and control groups, however, became higher 5 and 8 years after motor onset, respectively (Fig. 3C). Similarly, CSF p-tau level in PDCI group was estimated to be lower than that in the PDCU and control groups at the onset. However, temporal trajectory of CSF p-tau in PDCI group exhibited steadily increasing pattern (increase by 124% during 30 years after the onset) close to that of controls. In contrast, the estimated CSF p-tau level barely changed throughout the disease course in PDCU group (increase by only 3% during 30 years after the onset; Fig. 3D). Trajectories of serum NfL showed initial rapid rise and later deceleration pattern in both two groups. Serum NfL levels in both PDCU and PDCI groups were estimated to be higher than control group for 30 years after motor onset. PDCI group showed greater change in serum NfL level (285%) than PDCU group (217%; Fig. 3E). In both PDCU and PDCI groups, changes of z scores were most remarkable in serum NfL (Fig. 3F).

### Effect of CSF Aβ_1-42_ on temporal trajectories of biomarkers and cognitive outcomes

In the comparison of measured biomarker levels for 5 years, PD patients with low Aβ_1-42_ showed significantly lower CSF α-syn (estimate − 438.43, SE 83.41, *p* < 0.001), t-tau (estimate − 40.66, SE 7.21, *p* < 0.001) and p-tau levels (estimate − 2.64, SE 0.71, *p* < 0.001), whereas serum NfL did not exhibit significant difference (Supplementary Table S2). In addition, linear mixed effect models showed rapid decline of cognitive performance for 6-years of study period (interaction between group and disease duration; MoCA, estimate − 0.22, SE 0.03, *p* < 0.001; HVLT, estimate − 0.49, SE 0.15, *p* = 0.001; LNS, estimate − 0.07, SE 0.03, *p* = 0.023; Supplementary Table S3).

**Figure 4:**
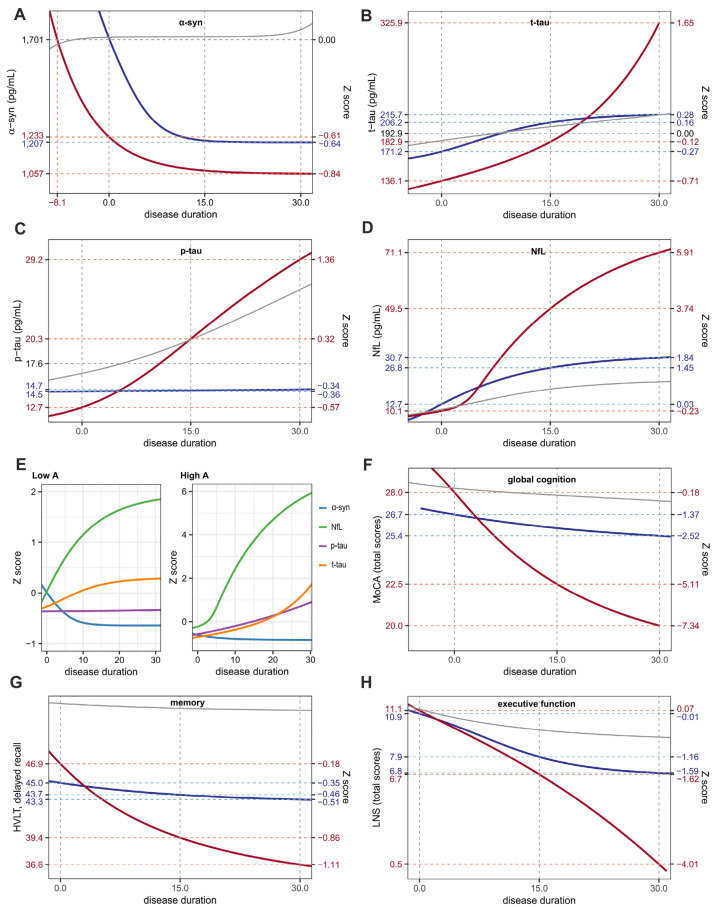
Temporal trajectories of biomarkers and cognitive performances in PD patients with high and low CSF Aβ_1-42_ at baseline. PD patients with low CSF Aβ_1-42_ levels (red lines) show greater change in four biomarkers (**A**: α-syn, **B**: t-tau, **C**: p-tau, **D**: NfL) and cognitive decline (**F**: global cognition, **G**: memory, H: executive function) across the disease course than those with high CSF Aβ_1-42_ (blue lines). NfL showed the greater changes in the temporal trajectory among biomarkers (**E**). Gray lines represent temporal trajectories in control group. Figures are illustrated using ggplot2 and patchwork packages in R software. *α-syn* α-synuclein; *t-tau* total tau; *p-tau* phosphorylated tau; *NfL* neurofilament light chain; *MoCA* Montreal Cognitive Assessment; *LNS* Letter-Number Sequencing test; *HVLT* Hopkins Verbal Learning Test.

Low Aβ_1-42_ group showed earlier reduction of CSF α-syn below Z-score 0 (8.1 years before the onset) than high Aβ_1-42_ group (0.1 years after the onset). As the trajectories of CSF α-syn approached to plateau, difference in CSF α-syn levels between two groups gradually decreased. However, low CSF Aβ_1-42_ group exhibited still lower estimated CSF α-syn level than that of the high CSF Aβ_1-42_ group (Fig. 4A). Although low CSF Aβ_1-42_ group showed lower estimated CSF t-tau and p-tau levels at baseline than high Aβ_1-42_ group, temporal trajectory of CSF tau proteins in low Aβ_1-42_ group showed rapid rise throughout the disease course (Fig. 4B and C), and eventually CSF t-tau and p-tau levels in low Aβ_1-42_ group became higher than those in the high Aβ_1-42_ group about 20 years (t-tau) and 5 years (p-tau) after motor onset. Compared to trajectories in control group, CSF t-tau in PD patients with low CSF Aβ_1-42_ became higher about 19 years after motor onset, whereas p-tau levels showed similar increase during estimated 30 years. For serum NfL, PD patients with high and low CSF Aβ_1-42_ exhibited rapid increase of biomarker level compared to controls. In high CSF Aβ_1-42_ group, estimated serum NfL level increased up to 30.7 pg/mL (Z-score = 1.84) for 30 years, while low CSF Aβ_1-42_ group showed steady increase in serum NfL level up to 71.1 pg/mL (Z-score = 5.91) at 30 years after the onset (Fig. 4D). Temporal trajectories of serum NfL showed higher z score changes for 30 years after motor onset compared to other biofluid markers (Fig. 4E).

Both groups showed similar cognitive performances at the onset of motor symptoms. However, low CSF Aβ_1-42_ group exhibited faster decline of cognitive performances than high Aβ_1-42_ group. During the course of 30 years of symptomatic period, there was a greater reduction in MoCA total score in low CSF Aβ_1-42_ group (− 28.6%) than that in high CSF Aβ_1-42_ group (− 4.8%; Fig. 4F). Compared to control group, PD patients had lower estimated HVLT delayed recall scores regardless of baseline CSF Aβ_1-42_ classification. HVLT delayed recall scores showed a greater reduction in low CSF Aβ_1-42_ (− 22.0%) than high CSF Aβ_1-42_ group (− 3.8%; Fig. 4G). Compared to the high CSF Aβ_1-42_ group, low CSF Aβ_1-42_ exhibited similar reduction of LNS total score during the first decade from the onset. However, after that time point, decline in high CSF Aβ_1-42_ group was attenuated, thus difference between both groups was increased up to 6.1 (Z score = 2.42) at 30 years after the onset (Fig. 4H).

## Discussion

Our temporal trajectory models exhibited that CSF Aβ_1-42_ and α-syn levels progressively decreased in a negative exponential pattern even before the onset of motor symptoms and approached a plateau. In contrast, CSF t-tau and p-tau, and serum NfL levels increased with the disease progression. These temporal changes in biomarkers were more likely to be greater in the PD patients with a cognitive impairment at baseline and in those with low CSF Aβ_1-42_ at baseline. PD patients with low CSF Aβ_1-42_ showed faster decline of cognitive performance than those with high CSF Aβ_1-42_ at baseline. Therefore, longitudinal changes in biomarkers can be influenced by the underlying cognitive impairment and amyloid-β pathology in PD.

We applied restricted cubic spline function to annual changes of biomarkers and integration of annual changes using ordinary differential equation modeling. These methods do not provide any statistical metrics, thus our analyses suggest a trend of further changes in biomarkers rather than a confirmative conclusion. Statistical analyses using empirical measurements would provide relatively conclusive results, however, as stated above, it is practically difficult to compose a cohort investigating long-term changes in biofluid markers. Moreover, statistical analysis requires a pre-defined assumption for shape of the trajectory. Without such a priori assumption, we attempted the mathematical model to predict long-term changes of biomarkers. Therefore, our study requires further cross-validation by longitudinal measurement of biofluid markers.

Previous studies investigating the longitudinal changes in CSF biomarkers in PD patients have reported inconsistent results^6–11,14^. An increase in CSF α-syn level was observed in a subset of PD patients selected by diagnostic likelihood over 90% in Deprenyl and Tocopherol Antioxidative Therapy for Parkinsonism (DATATOP) study^7^, whereas a contrary result was found in a study analyzing full dataset of the same cohort^8^. In PD without dementia, an increase in CSF α-syn level was observed only in the patients with disease duration longer than 5 years^11^. Moreover, contrary to our results, a previous analysis including 173 subjects PPMI database reported a significant increase in CSF Aβ_1-42_ during one year from baseline^10^. In our temporal trajectory models obtained from all PD patients in PPMI database, overall changes in CSF α-syn, Aβ_1-42_, t-tau and p-tau levels were only less than Z-score 1.0 during 30 years from the onset and more prominent in the patients with cognitive impairment and low CSF Aβ_1-42_. We suspect that the inconsistency between the previous studies was attributable to the relatively small long-term changes in CSF biomarkers and a heterogeneity of PD patients included. Indeed, a previous study including full dataset of PPMI demonstrated progressive decline of CSF Aβ1-42 during 3 years from baseline^15^. Therefore, a consistent and meaningful change can be expected by much longer observational period in a large number of PD patients with relatively homogeneous clinical features vulnerable to changes in CSF biomarkers.

Although the precise mechanism remains unclear, it was postulated that reduced CSF α-syn in PD may reflect altered dynamic of α-syn, possibly due to sequestration in intracellular LB pathology or enhanced extracellular clearance in α-synucleinopathy^16–18^. Nuclear imaging studies showed that decrease in CSF Aβ_1-42_ and increase in CSF t-tau and p-tau were correlated with increased amyloid and tau burden in brain^19,20^. Therefore, our results suggest that PD patients with cognitive impairment at early stage can be a phenotype resulting from an early appearance of LB load and rapid accumulation of amyloid and tau pathologies. LB and amyloid plaque pathologies in cortical and limbic regions are highly associated with the occurrence of dementia in the clinical course of PD^4,5,21^, and the patients with PD dementia were more likely to show lower CSF Aβ_1-42_ and higher CSF t-tau and p-tau levels, when compared to those with normal cognition^22–24^. Similar to our results, PD patients with low CSF Aβ_1-42_ at early stage showed worse cognitive performance^25,26^ and faster decline of cognitive performance in memory, executive and visuospatial function than those with high CSF Aβ_1-42_^27,28^.

NfL is a subunit of neurofilament protein, which is a major component of neuronal cytoskeleton and abundant in myelinated axons with large caliber^29,30^. Axonal degeneration preceding neuronal death and damaged white matter integrity in cellular model and patients with Alzheimer's disease are, for instance, the evidences for an involvement of white matter in neurodegenerative diseases^31–33^. Thus, CSF NfL level can be a biomarker for neuroaxonal damage in various kinds of neurodegenerative diseases^34^. A recent study showed a significant correlation between CSF and serum levels of NfL in PD patients, suggesting that serum NfL also can be a potential biomarker for neurodegeneration replacing CSF NfL^35^. Although some studies did not find an increased CSF NfL in PD^6,30,36^, the other studies observed an increased CSF or plasma NfL level in PD patients compared to the healthy elderly^35,37^. In a large biomarker study including over 3,000 subjects with dementia, patients with late onset AD or dementia with Lewy body (DLB) showed higher CSF NfL than PD subjects^30^. Therefore, we may expect that additional amyloid pathology in PD enhances an increase of NfL in both CSF and serum. It is interesting to note that estimated changes in serum NfL was much greater than CSF biomarkers in our study. Moreover, PD patients suspicious for having amyloid pathology exhibited great amount (almost 550%) of increase in serum NfL level over the 30 years. This suggests that the NfL level may be a potential biomarker for neurodegeneration induced by amyloid pathology^6^ or monitoring the disease progression in PD^37^.

It has been suggested that the dynamics of CSF α-syn, Aβ_1-42_, t-tau and p-tau may be intercorrelated in PD patients. Postmortem studies showed an association between cortical LB burden, amyloid plaque and tau grade^38,39^. Greater postmortem cortical LB burden was expected by the lower antemortem CSF Aβ_1-42_ level^40^. In transgenic mice model expressing cortical amyloid, early appearance of widespread α-syn pathology can be induced by an injection of preformed α-syn fibril into the hippocampus^41^. Similarly, α-syn pathology promoted fibrillization and phosphorylation of tau proteins in cellular and transgenic animal models^42,43^. Several studies demonstrated a correlation between CSF α-syn, Aβ_1-42_ and p-tau levels^6,25,44,45^. Therefore, amyloid load in central nervous system of PD patients may cause not only neurodegenerative process related with Alzheimer’s disease, but accelerated propagation of α-syn pathology. As stated above, our estimated trajectory of serum NfL suggested a possible impact of amyloid burden on neuroaxonal integrity, and disruption of white matter integrity are related to deterioration of cognitive performance in working memory, attention and executive function, visuospatial skills, and psychomotor speed^46^. Our estimated trajectories are consistent with previous studies showing predictive value of CSF Aβ1-42 for global and domain specific cognitive decline^15^.

In this study, we stratified PD group either by CSF Aβ_1-42_ or cognition at the time point of baseline assessment in PPMI database. Because the patients had various disease duration at baseline, our study can be limited by the absence of synchronization of CSF Aβ_1-42_ and cognition levels in dichotomizing groups. In addition, we extrapolated biomarker levels and cognitive outcomes about 30 years of disease duration using longitudinal measurement of 5–7 years. Because the PPMI database only included the PD patients in their early stage of disease course, our study is limited by missing biomarker data for the far advanced PD patients and the changes in biomarkers might be underestimated. Therefore, our temporal trajectory models require to be further revised by including advanced PD patients.

In summary, our temporal trajectory models suggest that longitudinal changes in biomarkers can vary with amyloid burden or cognitive impairment in PD patients. Early occurrence of cognitive impairment can be a clinical feature suspecting more rapid growth of LB and AD type pathologies. PD patients with low CSF Aβ_1-42_ may be more vulnerable to LB pathology, neuroaxonal damage, and cognitive impairment in their clinical course. Our study results raised a possibility for the potential role of future amyloid-lowering therapy for delaying the pathological progression and cognitive decline in PD.

## Methods

### Participants

**Figure 5:**
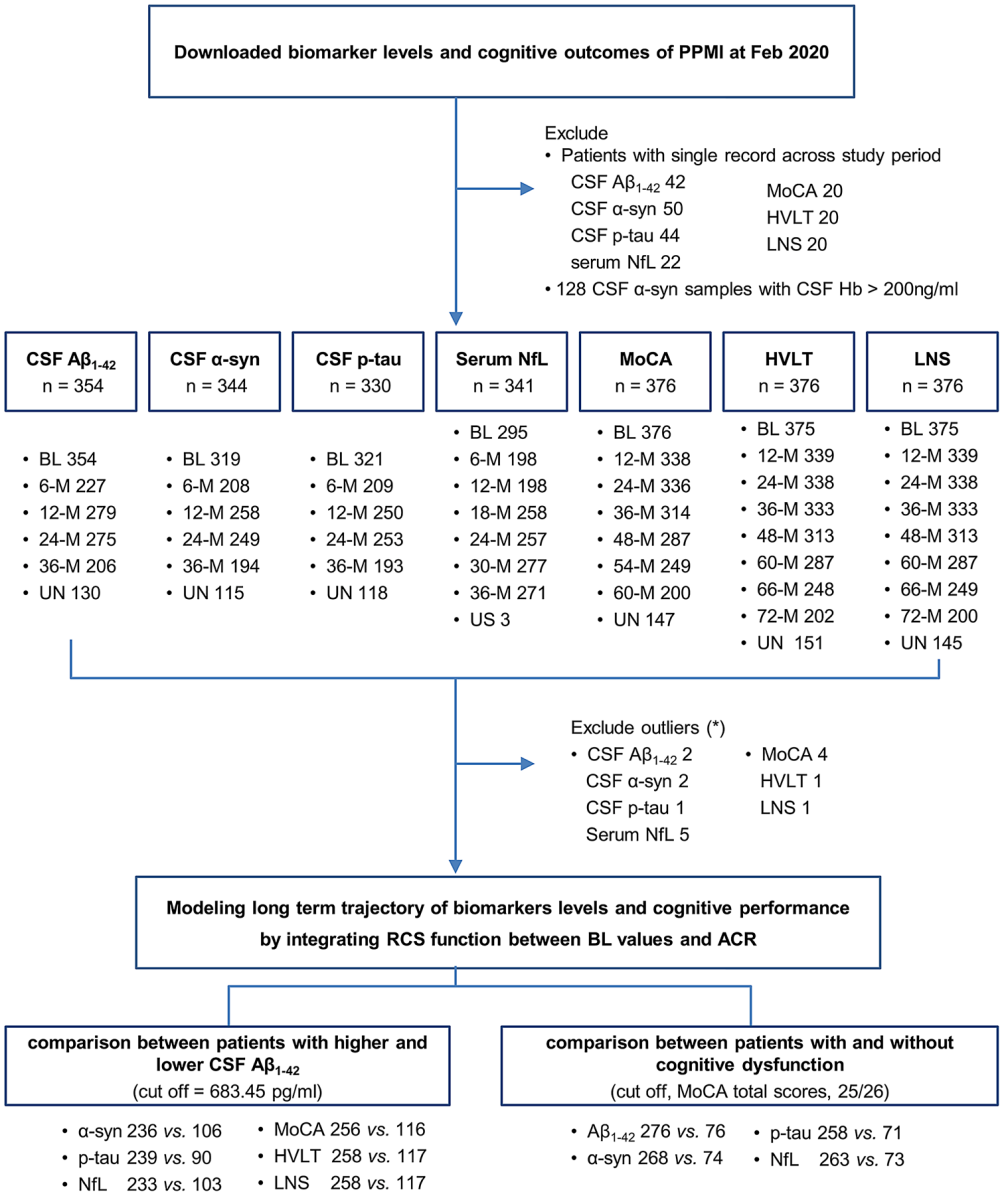
Study design. *Outliers were defined as patients with more than 3 interquartile ranges below the first quartile or above the third quartile in baseline biomarker levels, or patients with cognitive outcomes beyond the mean ± 4 SD. *PPMI* Parkinson's Progression Markers Initiative; *NfL* neurofilament light chain; *Hb* Hemoglobin; *MoCA* Montreal Cognitive Assessment, total scores; *HVLT* Hopkin's Verbal Learning test, total scores of delayed recall; *LNS* Letter-Number-Sequencing test, total scores; *BL* baseline; 6-, 12-, 18-, 24-, 30-, 36-, 48-, 54-, 60-, 66-, 72-M = months from baseline visit; *UN* unscheduled measurement; *ACR* annual change rates; *RCS* restricted cubic spline.

Study design is summarized in Fig. 5. From the PPMI database (http://ppmi-info.org), anonymized and de-identified results as of Feb 2020 were downloaded. Briefly, the PPMI study is an observational cohort study aimed at identifying biomarkers of PD progression. The eligibility criteria include PD patients older than 30 years of age, diagnosed with PD within the last 2 years with a Hoehn & Yahr (H&Y) stage not greater than II, untreated, and exhibiting striatal dopaminergic dysfunction on ^123^I-N-3-fluoropropyl-2β-carbomethoxy-3β-4-iodophenyl tropane (^123^I-FP-CIT) single photon emission computed tomography (SPECT). The detailed study protocol can be found at www.ppmi-info.org.

We included PD and control subjects who had undergone measurement of CSF α-syn, Aβ_1-42_, p-tau and serum NfL at least twice. Age-at-onset (AAO) was defined as the time interval between date of birth and self-declared motor onset, and disease duration as the time interval between the self-declared motor onset and sampling date. The severity of parkinsonian motor deficits was assessed by the Movement Disorder Society sponsored Unified Parkinson Disease Rating Scale (MDS-UPDRS) part III and H&Y stage. To estimate the effect of baseline CSF Aβ_1-42_ on longitudinal cognitive performance, we included total scores of Montreal Cognitive Assessment (MoCA), Letter-Number Sequencing (LNS) test, and delayed recall scores of Hopkins Verbal Learning Test (HVLT). In addition, genotyping of Apolipoprotein E (APOE) was included in the analyses. We classified PD patients into cognitively unimpaired (PDCU, MoCA total scores > 25) and cognitively impaired (PDCI, MoCA total scores ≤ 25) groups^47^.

The PPMI study is registered at ClinicalTrials.gov (NCT01141023). Each PPMI site received approval from an ethics committee on human experimentation prior to study initiation. All research was performed in accordance with the relevant guidelines and regulations. The institutional review board of Pusan National University approved secondary analyses of publically shared data. The informed written consent has been waived off by the institutional review board of Pusan National University.

### Measurement of biomarkers

CSF was collected by standardized lumbar puncture procedures. Shipment and storage were performed as described in the PPMI biological manual (http://ppmi-info.org) and elsewhere^26,48^. The coded frozen aliquots of CSF were transferred from the PPMI Biorepository Core laboratories to the University of Pennsylvania and to Covance for analyses. CSF Aβ_1-42_, t-tau and p-tau were measured using the electrochemiluminescence (ECL) immunoassays on a fully automated cobas e601 analyzer (Elecsys, Roche diagnostic). The concentration of α-syn in CSF samples were analyzed using commercially available sandwich type ELISA kits (Covance, Dedham, MA), as previously described^26^. Serum NfL level was measured by the 2-step digital immunoassay using Single Molecule Array (Simoa) technology (NF-light; UmanDiagnostics, Umeå, Sweden). In the present study, CSF α-syn samples with low hemoglobin (< 200 ng/mL)^9^ were included in the analyses. As proposed by a recent study analyzing PPMI database^15^, we used cut-off 683.45 pg/mL for deciding high and low Aβ_1-42_ groups. In addition, we excluded the PD patients with baseline biomarker levels more than 3 interquartile ranges below first quartile or above the third quartile.

### Estimation of temporal trajectories

As described in the previous literatures^12,13^, we first applied linear regression for each subject to calculate the annual change rates of CSF biomarker levels over time. Linear regression model of each subject had disease duration (year) as predictor and biomarker or scores of cognitive function tests as responder variable. In case of control group, time interval from baseline (year) was used as predictor term. In addition, CSF biomarker levels and scores of cognitive function tests at the onset of motor symptoms were calculated using the linear regression model. Then, we acquired a curve for annual changes in each biomarker as a function of baseline values by using restricted cubic spline with 4 knots. The knots were placed at 5-, 35-, 65- and 95-percentile values of baseline levels. When sample size was under 100, we applied 3 knots at 5-, 50-, 95-percentile values. Estimated levels as a function of time in year were acquired by using the modified Euler’s method for solving the first order differential equation. Finally, based on the calculated biomarker levels at motor onset, we plotted trajectories of estimated CSF biomarker levels between the time points at 5 years before and 30 years after the onset of motor symptoms. To compare with control subjects, we also calculated Z-score using the mean and SD obtained from baseline values of control groups. In addition, we estimated temporal trajectories of biomarkers and scores of cognitive function tests in control group using same method. Calculated trajectories of control group were anchored to time axis (age) using mean values of biomarkers and cognitive performances and median age at baseline visit of PPMI study. For comparison between PD and control groups, we converted age into disease duration using following formula: disease duration = age – median of age at motor onset in PD patients.

### Statistical analyses

Age, disease duration, education period and MDS-UPDRS III total scores were compared using independent t-test. Chi-square test was employed to compare categorical variables including sex ratio, APOE e4 allele frequency, and H&Y stage. Comparison of longitudinal biomarker levels and cognitive outcomes between groups were testing by linear mixed effect models using group, disease duration, and interaction between group and disease duration as fixed effects and subjects as random effect. Biomarker levels and cognitive outcomes at baseline were tested using generalized linear model covariated with age and sex. Statistical significance was defined as *p* < 0.05. Estimation of temporal trajectories and statistical analyses were conducted by custom script written in R software (version 3.6.2; r-project.org) with Hmisc and rms packages.

## Supplementary Information

Supplementary Information.

## Data Availability

The full dataset from the PPMI study is available at www.ppmi-info.org.
