# Supplementary material for: Temporal trajectory of biofluid markers in Parkinson’s disease
Source: Sci Rep. 2021 Jul 20;11:14820. doi: 10.1038/s41598-021-94345-8 (PMC8292456; doi:10.1038/s41598-021-94345-8)
Supplement: Supplementary file 1 — Supplementary Information. [file 41598_2021_94345_MOESM1_ESM.docx]

**[Supplementary information]**

**Temporal trajectory of biofluid markers in Parkinson’s disease**

Min Seok Baek,^1,3^ Myung Jun Lee,^2*^ Han-Kyeol Kim,^3^ Chul Hyoung Lyoo,^3^

^1^ Department of Neurology, Wonju Severance Christian Hospital, Yonsei University Wonju

College of Medicine, Wonju, Gangwon do, Republic of Korea

^2^ Department of Neurology, Pusan National University Hospital, Pusan National University School of Medicine and Biomedical Research Institute, Gudeok-ro 179, Seo-gu, Busan, Republic of Korea

^3^ Department of Neurology, Gangnam Severance Hospital, Yonsei University College of Medicine, 20 Eonjuro 63-gil, Gangnam-gu, Seoul, Republic of Korea

*** Correspondence:**

Myung Jun Lee, MD

Clinical associate professor

Department of Neurology

Pusan National University Hospital

Pusan National University School of Medicine and Biomedical Research Institute

Gudeok-ro 179, Seo-gu, Busan, 49241, Republic of Korea

Tel: +82-51-240-7317 Fax: +82-51-245-2783

**Character count for Title**: 62

**Table** 1; **Figures** 5; **References** 48

**Word count for Abstract**: 200; **Word count for Text**: 3478

**Supplementary Table 1. Comparison of measured biofluid marker levels between PDCU and PDCI groups**

|  |  | Baseline | 6-month | 1-year | 2-year | 3-year | 5-year |  | E (*p*) |
| --- | --- | --- | --- | --- | --- | --- | --- | --- | --- |
| Aβ_1-42_ | CU | 907.2 ± 381.2 | 936.5 ± 400.2 | 888.6 ± 360.1 | 878.6 ± 350.4 | 890.7 ± 335.3 | - | G | -0.95 (0.986) |
|  | CI | 934.9 ± 416.8 | 948.7 ± 416.5 | 980.0 ± 491.6 | 933.0 ± 473.0 | 935.4 ± 451.2 | - | G×D | 22.94 (0.016) |
| α-syn | CU | 1488.0 ± 605.8 | 1530.4 ± 829.4 | 1395.0 ± 568.6 | 1408.1 ± 539.0 | 1436.1 ± 567.5 | - | G | -138.54 (0.144) |
|  | CI | 1527.7 ± 678.2 | 1477.4 ± 687.8 | 1523.6 ± 717.0 | 1527.0 ± 757.5 | 1483.8 ± 677.4 | - | G×D | 63.31 (< 0.001) |
| t-tau | CU | 167.1 ± 52.3 | 168.3 ± 55.7 | 162.4 ± 53.6 | 166.0 ± 58.7 | 168.3 ± 56.9 | - | G | -1.93 (0.817) |
|  | CI | 173.3 ± 64.4 | 180.1 ± 76.7 | 178.3 ± 64.9 | 180.6 ± 70.1 | 189.2 ± 72.6 | - | G×D | 4.41 (< 0.001) |
| p-tau | CU | 14.6 ± 4.8 | 14.8 ± 4.7 | 14.4 ± 4.9 | 14.5 ± 5.3 | 14.7 ± 5.3 | - | G | 0.23 (0.762) |
|  | CI | 15.8 ± 6.2 | 16.3 ± 7.0 | 16.0 ± 6.2 | 16.1 ± 6.6 | 16.2 ± 6.4 | - | G×D | 0.34 (0.001) |
| NfL | CU | 12.8 ± 8.7 | 13.9 ± 9.9 | 14.3 ± 12.2 | 15.6 ± 13.1 | 15.6 ± 10.3 | 18.6 ± 16.2 | G | 0.74 (0.684) |
|  | CI | 14.3 ± 6.3 | 16.7 ± 9.1 | 18.1 ± 18.7 | 17.2 ± 9.0 | 19.1 ± 13.8 | 21.9 ± 22.2 | G×D | 0.43 (0.159) |

Mean ± SD. Abbreviation: Aβ_1-42_ = amyloid-β_1-42_; α-syn = α-synuclein; t-tau = total tau; p-tau = phosphorylated tau; NfL = neurofilament light chain; CU = cognitively unimpaired; CI = cognitively impaired; E = estimates of linear mixed effect model using group (CU and CI), disease duration (years) and interaction between group and disease duration as fixed effect and subject as random effect; *p* = p-values in linear mixed effect models; G = group effect in linear mixed effect models; G×D = effect of interaction between group and disease duration in linear mixed effect models. Values in red color = statistically significant.

**Supplementary Table 2. Comparison of measured biofluid marker levels between PD groups with high and low CSF Aβ_1-42_.**

|  | **α-syn** | |  | **t-tau** | |  | **p-tau** | |  | **NfL** | |
| --- | --- | --- | --- | --- | --- | --- | --- | --- | --- | --- | --- |
|  | high Aβ_1-42_ | low Aβ_1-42_ |  | high Aβ_1-42_ | low Aβ_1-42_ |  | high Aβ_1-42_ | low Aβ_1-42_ |  | high Aβ_1-42_ | low Aβ_1-42_ |
| Baseline | 1645.8 ± 614.2 | 1182.2 ± 509.5 |  | 179.0 ± 50.4 | 143.5 ± 57.8 |  | 15.3 ± 4.7 | 13.4 ± 5.9 |  | 13.0 ± 8.0 | 13.6 ± 8.7 |
| 6-month | 1629.4 ± 824.8 | 1212.3 ± 633.9 |  | 181.9 ± 59.6 | 141.1 ± 54.0 |  | 15.6 ± 5.2 | 13.4 ± 5.3 |  | 14.5 ± 10.6 | 14.1 ± 7.3 |
| 1-year | 1563.0 ± 596.8 | 1142.4 ± 524.4 |  | 174.9 ± 51.2 | 146.9 ± 63.2 |  | 15.0 ± 4.8 | 14.1 ± 6.5 |  | 14.6 ± 14.2 | 16.3 ± 13.0 |
| 2-year | 1586.1 ± 584.4 | 1123.2 ± 490.9 |  | 179.9 ± 55.5 | 146.4 ± 67.7 |  | 15.4 ± 5.0 | 13.5 ± 6.9 |  | 15.1 ± 11.1 | 17.8 ± 14.4 |
| 3-year | 1590.0 ± 555.1 | 1132.9 ± 541.2 |  | 181.5 ± 53.1 | 151.5 ± 71.0 |  | 15.4 ± 4.7 | 13.8 ± 7.3 |  | 16.1 ± 10.7 | 17.1 ± 12.4 |
| 5-year | - | - |  | - | - |  | - | - |  | 18.9 ± 15.8 | 20.4 ± 21.6 |
| **Linear mixed effect models** | |  |  |  |  |  |  |  |  |  |  |
|  | β (SE) | *p* |  | β (SE) | *p* |  | β (SE) | *p* |  | β (SE) | *p* |
| group | -438.43 (83.41) | < 0.001 |  | -40.66 (7.21) | < 0.001 |  | -2.64 (0.71) | < 0.001 |  | 0.74 (1.82) | 0.685 |
| interaction | -1.99 (18.19) | 0.913 |  | 1.99 (7.21) | 0.093 |  | 0.17 (0.10) | 0.085 |  | 0.43 (0.30) | 0.159 |

Mean ± SD; SE = standard error. β = estimate of linear mixed effect models using group (high and low Aβ_1-42_), disease duration (year) and interaction between group and disease duration, and subject as random effect; Aβ_1-42_ = amyloid-β_1-42_; α-syn = α-synuclein; t-tau = total tau; p-tau = phosphorylated tau; NfL = neurofilament light chain; numbers in red color = statistically significant (*p* < 0.05).

**Supplementary Table 3. Comparison of neuropsychiatric performances between PD groups with high and low CSF Aβ_1-42_.**

|  | **MoCA** | |  | **HVLT** | |  | **LNS** | |
| --- | --- | --- | --- | --- | --- | --- | --- | --- |
|  | high Aβ_1-42_ | low Aβ_1-42_ |  | high Aβ_1-42_ | low Aβ_1-42_ |  | high Aβ_1-42_ | low Aβ_1-42_ |
| Baseline | 27.0 ± 2.3 | 27.3 ± 2.3 |  | 44.6 ± 11.0 | 44.9 ± 11.5 |  | 10.5 ± 2.7 | 10.6 ± 2.5 |
| 1-year | 26.4 ± 2.6 | 26.2 ± 3.3 |  | 44.4 ± 11.7 | 44.2 ± 12.7 |  | 10.5 ± 2.6 | 10.1 ± 2.6 |
| 2-year | 26.5 ± 2.7 | 25.7 ± 3.8 |  | 45.7 ± 11.3 | 44.3 ± 12.9 |  | 10.5 ± 2.6 | 9.7 ± 3.0 |
| 3-year | 26.5 ± 2.8 | 26.2 ± 3.3 |  | 46.7 ± 11.5 | 45.4 ± 13.2 |  | 10.3 ± 2.9 | 9.7 ± 3.2 |
| 4-year | 26.8 ± 3.1 | 25.8 ± 4.0 |  | 47.0 ± 11.9 | 42.0 ± 13.8 |  | 10.2 ± 3.0 | 9.6 ± 3.5 |
| 5-year | 26.6 ± 3.2 | 26.1 ± 4.5 |  | 48.5 ± 12.3 | 45.3 ± 13.2 |  | 10.1 ± 2.9 | 9.7 ± 3.3 |
| 6-year | 26.7 ± 3.6 | 26.1 ± 4.2 |  | 46.3 ± 12.7` | 42.5 ± 13.7 |  | 9.8 ± 2.9 | 9.2 ± 2.9 |
| **Linear mixed effect moddel** | |  |  |  |  |  |  |  |
|  | β (SE) | *p* |  | β (SE) | *p* |  | β (SE) | *p* |
| group | 0.56 (0.34) | 0.102 |  | 0.64 (1.34) | 0.635 |  | -0.12 (0.32) | 0.722 |
| interaction | -0.22 (0.03) | < 0.001 |  | -0.49 (0.15) | 0.001 |  | -0.07 (0.03) | 0.023 |

Mean ± SD; SE = standard error. β = estimate of linear mixed effect models using group (high and low Aβ_1-42_), disease duration (year) and interaction between group and disease duration, and subject as random effect; MoCA = Montreal Cognitive Assessment, total scores; HVLT = Hopkins Verbal Learning Tests, delayed recall scores; LNS = Letter Number Sequencing, total scores; Aβ_1-42_ = amyloid-β_1-42_; α-syn = α-synuclein; t-tau = total tau; p-tau = phosphorylated tau; NfL = neurofilament light chain; numbers in red color = statistically significant (*p* < 0.05).

**
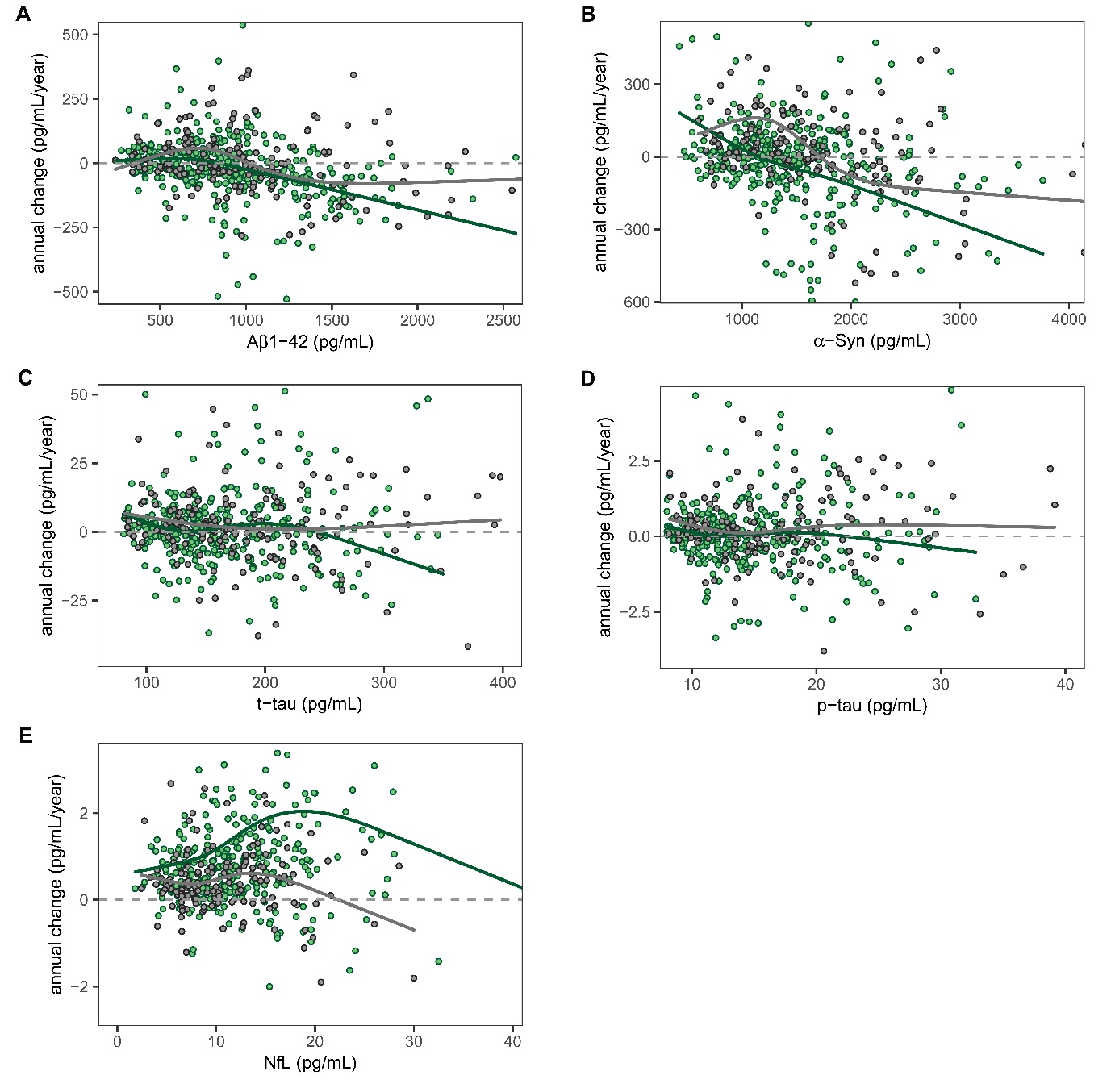
**

**Supplementary Figure 1. Annual changes in biomarkers as a function of their baseline levels.** Fitted curves on the scatter plots were obtained with restricted cubic spline model. Green dots and lines represent annual changes and restricted cubic spline function in PD group, whereas gray dots and lines indicate those in controls.
